# Periodontal outcomes: Surgical exposure versus extraction of impacted maxillary canines

**DOI:** 10.6026/973206300222833

**Published:** 2026-05-31

**Authors:** Tarun Choudhary, Sardhar Malothu, Archisman Mukherjee, Manali Prakash Jadhav, Ritik Roshan, Geetu Singh

**Affiliations:** 1 Department of Oral and Maxillofacial Surgery, Government Dental College, Jodhpur, Rajasthan, India; 2 Department of Dentistry, Father Colombo Medical College and Hospital, Warangal, Telangana, India; 3 Department of Periodontology, Awadh Dental College and Hospital, Jamshedpur, Jharkhand, India; 4 Department of Orthodontics and Dentofacial Orthopaedics, Government Dental College & Hospital, Jamnagar, Gujarat, India; 5 Department of Orthodontics & Dentofacial Orthopaedics, Awadh Dental College and Hospital, Jamshedpur, Jharkhand, India; 6 Department of Oral & Maxillofacial Surgery, Maharishi Markendshwar College of Dental Sciences & Research, Maharishi Markendshwar (Deemed to be University), Mullana, Haryana, India

**Keywords:** Impacted maxillary canine, orthodontic traction, periodontal outcomes, surgical exposure, tooth extraction

## Abstract

Impacted maxillary canines pose a clinical dilemma in selecting an optimal treatment modality while preserving periodontal health. Therefore, it is of interest to evaluate periodontal outcomes between surgical exposure with orthodontic traction and extraction in 100 patients over 12 months. Indices assessed included plaque index (PI), gingival index (GI), probing pocket depth (PPD), clinical attachment loss, gingival recession and alveolar bone levels. Results showed significantly greater increases in PPD, CAL, GR and bone loss in the surgical exposure group, while extraction maintained relatively stable periodontal parameters (p < 0.05). Thus, we show integrating periodontal risk assessment into treatment planning for impacted maxillary canines.

## Background:

Impacted maxillary canines represent one of the most common and clinically significant eruption disturbances encountered in dental practice, second only to third molars. Their strategic position in the dental arch contributes not only to esthetics but also to functional occlusion, arch stability and guidance of mandibular movements [1]. Failure of eruption of these teeth can result in a range of complications, including root resorption of adjacent incisors, cyst formation, periodontal defects and esthetic concerns. The management of impacted maxillary canines remains a challenging clinical scenario that requires a multidisciplinary approach involving orthodontics, oral surgery and periodontics [2]. The etiology of canine impaction is multifactorial, encompassing genetic predisposition, guidance theory (absence or malformation of lateral incisors), arch length deficiency, abnormal tooth germ position and local pathological conditions [3]. Early diagnosis through clinical and radiographic examination plays a crucial role in preventing complications and improving treatment outcomes. Radiographic modalities such as panoramic radiographs and cone-beam computed tomography (CBCT) have significantly enhanced the ability to accurately locate impacted canines and assess their relationship with adjacent structures [4]. Various treatment modalities have been proposed for managing impacted maxillary canines, broadly categorized into surgical exposure followed by orthodontic traction and extraction of the impacted tooth [5]. Surgical exposure with orthodontic alignment is often considered the treatment of choice, especially in younger patients, as it allows preservation of the natural tooth and maintenance of alveolar bone integrity. Techniques such as open and closed eruption methods are commonly employed, each with its own advantages and limitations. Successful orthodontic alignment depends on factors such as the position and angulation of the impacted tooth, patient age and available space in the dental arch [6]. However, surgical exposure combined with orthodontic traction is not without potential drawbacks. It may involve prolonged treatment duration, increased patient discomfort, risk of ankylosis and possible periodontal complications such as gingival recession (GR), loss of attachment and bone defects [7].

The periodontal health of the aligned canine is a critical consideration, as compromised periodontal support can affect long-term prognosis and esthetic outcomes. Studies have reported varying degrees of periodontal changes following orthodontic traction, highlighting the need for careful surgical technique and optimal orthodontic force application [8]. On the other hand, extraction of impacted maxillary canines is often considered in cases where the tooth is severely displaced, ankylosed, or associated with pathology, or when orthodontic alignment is deemed unfeasible [9]. Extraction may provide a simpler and shorter treatment option, especially in adult patients or those unwilling to undergo prolonged orthodontic therapy. Following extraction, treatment options include space closure with orthodontic movement of adjacent teeth or prosthetic replacement using implants or bridges. While extraction eliminates the risks associated with surgical exposure and traction, it may lead to alveolar bone loss, altered o cclusion and compromised esthetics if not managed appropriately [10]. Despite the availability of multiple treatment options, there is still a lack of consensus regarding the optimal approach for managing impacted maxillary canines, particularly in terms of periodontal health. Existing literature presents conflicting results, with some studies favoring surgical exposure and orthodontic alignment due to its conservative nature, while others highlight the potential benefits of extraction in selected cases [11]. Furthermore, variations in surgical techniques, orthodontic protocols and patient-related factors contribute to the heterogeneity of outcomes reported in different studies. Given the increasing emphasis on evidence-based clinical decision-making, it is imperative to systematically compare the periodontal outcomes associated with these treatment modalities [12]. Therefore, it is of interest to describe the comparative periodontal outcomes following surgical exposure with orthodontic traction versus extraction of impacted maxillary canines.

## Methodology:

This prospective comparative clinical study was conducted at a tertiary dental care institution over a period of 18-24 months, encompassing patient recruitment, treatment and follow-up evaluation. A total of 100 patients diagnosed with impacted maxillary canines were selected from the outpatient department based on predefined inclusion and exclusion criteria and were equally allocated into two groups: Group A (n = 50), treated with surgical exposure followed by orthodontic traction and Group B (n = 50), managed by extraction of the impacted canines. Eligible participants were aged between 15-30 years, presented with unilateral or bilateral impacted maxillary canines confirmed clinically and radiographically were systemically healthy and provided informed consent, while individuals with systemic diseases affecting periodontal health, prior orthodontic treatment involving maxillary canines, craniofacial anomalies, severe baseline periodontal disease, or tobacco use were excluded. Ethical approval was obtained from the Institutional Ethical Committee and the study adhered to established ethical guidelines for human research. All patients underwent comprehensive clinical and radiographic evaluation, including panoramic radiographs and, where indicated, CBCT to assess the position, angulation and relationship of impacted canines with adjacent structures. In Group A, surgical exposure was performed using open or closed eruption techniques based on clinical judgment, followed by bonding of an orthodontic attachment and application of controlled orthodontic forces using fixed appliance therapy to guide the canine into proper alignment. In Group B, surgical extraction of the impacted canine was carried out under local anesthesia, followed by appropriate orthodontic space management or prosthetic rehabilitation depending on individual treatment requirements. Periodontal parameters, including plaque Index (PI), gingival Index (GI), probing pocket depth (PPD), clinical attachment level (CAL), GR and width of keratinized gingiva (WKG), were recorded at baseline, 6 months and 12 months using a standardized periodontal probe. All measurements were obtained at six sites per tooth by a single calibrated examiner to ensure consistency. Radiographic assessment of alveolar bone levels was performed using standardized intraoral periapical radiographs, with measurements taken from the cemento-enamel junction (CEJ) to the alveolar crest using digital software for enhanced accuracy. Examiner calibration was conducted prior to the study and intra-examiner reliability demonstrated strong agreement (k > 0.80). Data were systematically tabulated and analyzed using Statistical Package for the Social Sciences SPSS) version 25.0. Descriptive statistics, including mean and standard deviation, were calculated, while intra-group comparisons were performed using paired t-tests and inter-group comparisons using independent t-tests, with a p-value of <0.05 considered statistically significant. The primary outcome measure was periodontal health status assessed through clinical and radiographic parameters, while secondary outcomes included treatment duration and associated complications of each treatment modality.

## Results:

A total of 100 patients with impacted maxillary canines were included in the study and equally divided into two groups: Group A (surgical exposure with orthodontic traction) and Group B (extraction of impacted canines). All patients completed the study period and no significant dropouts were reported. The periodontal parameters were assessed at baseline, 6 months and 12 months. At baseline, there was no statistically significant difference between the two groups in terms of age, gender distribution, or periodontal parameters (p > 0.05), indicating comparability between the groups (Table 1). Group A showed a statistically significant increase in PPD and CAL at 6 and 12 months compared to baseline (p < 0.05). In contrast, Group B demonstrated relatively stable periodontal parameters with minimal changes over time (Table 2). At 12 months, Group A exhibited significantly higher PPD and CAL values compared to Group B (p <0.05), indicating comparatively compromised periodontal outcomes in the surgical exposure group (Table 3). Radiographic analysis revealed a greater reduction in alveolar bone height in Group A compared to Group B at 12 months, which was statistically significant (p < 0.05) (Table 4). Overall, patients treated with surgical exposure and orthodontic traction (Group A) demonstrated significantly greater deterioration in periodontal parameters compared to those undergoing extraction (Group B). These findings suggest that while surgical exposure preserves the natural tooth, it may be associated with compromised periodontal health if not carefully managed.

## Discussion:

The present study aimed to comparatively evaluate periodontal outcomes following surgical exposure with orthodontic traction versus extraction of impacted maxillary canines. The findings demonstrated that patients treated with surgical exposure and orthodontic traction exhibited significantly greater PPD, CAL, GR, CAL loss compared to those undergoing extraction. These results suggest that although orthodontic alignment preserves the natural tooth, it may compromise periodontal health if not carefully managed. The increased PPD and CAL observed in Group A in the present study are consistent with findings reported by Seehra *et al.* (2023) [11] who conducted a systematic review evaluating periodontal outcomes following surgical exposure and orthodontic alignment of impacted canines. Their study concluded that aligned impacted teeth often present with increased PPD and CAL compared to normally erupted teeth, highlighting the potential periodontal risks associated with traction procedures. Similarly, the greater alveolar bone loss observed in the orthodontic traction group in the present study is in agreement with Tarkan and Gürbüz (2026) [12] who reported that impacted canine treatment involving orthodontic traction is associated with significantly increased alveolar bone loss and longer treatment duration. Their study emphasized that mechanical forces applied during traction may adversely affect surrounding bone and periodontal structures. The present study also demonstrated that periodontal deterioration was more pronounced over time in the traction group, which aligns with the findings of Mousa *et al.* (2023) [13]. In their randomized controlled trial, they compared periodontal outcomes of impacted canines treated with different traction methods and found that periodontal parameters such as probing depth and gingival condition can worsen following orthodontic traction, depending on technique and duration of treatment. Furthermore, the findings of increased GR and compromised periodontal health in the traction group are supported by de Araújo *et al.* (2020) [14] who conducted a meta-analysis on surgical-periodontal aspects of orthodontic traction. They reported that impacted canines treated with surgical exposure and traction often show higher levels of GR and periodontal discrepancies when compared to contralateral teeth, suggesting that surgical and orthodontic manipulation can affect soft tissue stability. In addition, the role of initial impaction severity in influencing periodontal outcomes observed indirectly in the present study is comparable to findings by Caprioglio *et al.* (2019) [15] who reported that more severely displaced canines tend to exhibit poorer periodontal outcomes after alignment. Their study highlighted that factors such as depth and angulation of impaction significantly influence the extent of periodontal compromise following treatment. In contrast, the extraction group in the present study demonstrated relatively stable periodontal parameters with minimal changes over time. This may be attributed to the elimination of surgical exposure and orthodontic traction forces, thereby reducing trauma to periodontal tissues. However, it is important to note that while extraction may preserve periodontal health locally, it may lead to other concerns such as alveolar ridge resorption and need for prosthetic rehabilitation, which were not the primary focus of this study. The differences observed between the two groups in the present study can be explained by several biological and mechanical factors. Surgical exposure disrupts the soft tissue and bone architecture, while orthodontic traction applies continuous forces that may induce inflammatory responses, bone remodeling and potential attachment loss. Additionally, plaque accumulation around orthodontic appliances may further exacerbate periodontal inflammation, contributing to worsening clinical parameters. Overall, the findings of the present study are in agreement with the majority of existing literature, which suggests that orthodontic traction of impacted maxillary canines, although beneficial in preserving natural dentition, may be associated with compromised periodontal outcomes when compared to extraction. Therefore, careful consideration of periodontal implications is essential when selecting the appropriate treatment modality.

## Conclusion:

We show that surgical exposure with orthodontic traction of impacted maxillary canines is associated with greater periodontal deterioration, evidenced by increased PPD, CAL and GR. In contrast, extraction of impacted canines showed relatively stable periodontal outcomes over the study period. These findings underscore the importance of careful case selection and emphasize those periodontal considerations should play a decisive role in determining the optimal treatment modality.

## Figures and Tables

**Table 1:** Baseline demographic and clinical characteristics of study population

**Parameter**	**Group A (n=50)**	**Group B (n=50)**	**p-value**
Mean Age (years)	21.4 ± 3.2	22.1 ± 3.6	0.312
Male/Female	24/26	22/28	0.689
PI	1.21 ± 0.30	1.18 ± 0.28	0.621
GI	1.09 ± 0.25	1.05 ± 0.27	0.534
PPD (mm)	2.31 ± 0.41	2.28 ± 0.39	0.711
CAL	2.45 ± 0.38	2.41 ± 0.36	0.648

**Table 2:** Intra-group comparison of periodontal parameters over time

**Parameter**	**Time Point**	**Group A (Mean ± SD)**	**p-value**	**Group B (Mean ± SD)**	**p-value**
PPD (mm)	Baseline	2.31 ± 0.41		2.28 ± 0.39	
	6 months	2.78 ± 0.46	0.001*	2.35 ± 0.42	0.082
	12 months	3.12 ± 0.52	0.000*	2.41 ± 0.40	0.065
CAL (mm)	Baseline	2.45 ± 0.38		2.41 ± 0.36	
	6 months	2.96 ± 0.44	0.002*	2.50 ± 0.39	0.074
	12 months	3.28 ± 0.49	0.000*	2.56 ± 0.41	0.069
(*Statistically significant)

**Table 3:** Inter-group comparison of periodontal parameters at 12 months

**Parameter**	**Group A (Mean ± SD)**	**Group B (Mean ± SD)**	**p-value**
PI	1.36 ± 0.32	1.22 ± 0.29	0.041*
GI	1.28 ± 0.30	1.15 ± 0.27	0.038*
PPD (mm)	3.12 ± 0.52	2.41 ± 0.40	0.000*
CAL (mm)	3.28 ± 0.49	2.56 ± 0.41	0.000*
GR (mm)	0.82 ± 0.20	0.45 ± 0.18	0.001*

**Table 4:** : Radiographic assessment of alveolar bone loss

**Parameter**	**Group A (Mean ± SD)**	**Group B (Mean ± SD)**	**p-value**
Bone Loss (mm) at 12 months	1.42 ± 0.36	0.84 ± 0.28	0.000*
